# Challenges in diagnosis and management of xanthogranulomatous salpingo-oophoritis: A rare case

**DOI:** 10.1016/j.ijscr.2024.110778

**Published:** 2024-12-24

**Authors:** Mahalakshmi Gaddi, Sahana Surpur, Vidya Kamat, Zoha Khan, Mayur Wanjari, Gaurav Mittal

**Affiliations:** a Department of Obstetrics and Gynecology, SDM College of Medical Sciences and Hospital, Dharwad, India; b Azad Jammu Kashmir Medical College, Muzaffarabad, AJK, Pakistan; c Department of Research and Development, Datta Meghe Institute of Higher Education and Research Development, Wardha, India; d Department of Obstetrics and Gynecology, Mahatma Gandhi Institute of Medical Sciences, Wardha, India

**Keywords:** Xanthogranulomatous salpingo-oophoritis, Case report, Tubo-ovarian abscess, Pelvic malignancy mimic, Misdiagnosis

## Abstract

**Introduction and importance:**

Xanthogranulomatous inflammation of the female genital tract is a rare condition involving ovarian tubes characterized by chronic inflammation and destruction of pelvic organs, often mimicking pelvic malignancy.

**Case presentation:**

A 37-year-old female with a history of chronic kidney disease, hypertension, and treated pulmonary tuberculosis, presented with lower abdominal fullness, pain, and irregular menstrual cycles.

**Clinical discussion:**

Radiological investigations revealed a significant left adnexal mass, suggesting a tubo-ovarian abscess or neoplastic lesion. Staging laparotomy was performed, and intraoperatively, the mass was found adherent to surrounding organs. A frozen section analysis indicated a benign lesion and a hysterectomy was deferred. Histopathological evaluation confirmed the diagnosis of xanthogranulomatosis salpingo-oophoritis.

**Conclusion:**

This case underscores the importance of considering rare inflammatory conditions in the differential diagnosis of pelvic masses. It highlights the challenges in management, including the risk of extensive surgeries leading to infertility. Thorough investigation and accurate diagnosis are crucial for appropriate management and to minimize unnecessary interventions.

## Highlights

• Challenges in diagnosis and management highlighted
• Novel histopathological analysis for accurate diagnosis
• Comprehensive assessment guiding appropriate surgical intervention
• Emphasis on fertility preservation strategies
• Importance of ongoing monitoring and management for optimal patient care

## 1 Introduction

Xanthogranulomatous inflammation of the female genital tract is a rare pathological entity characterized by chronic inflammation and destruction of pelvic organ tissues, primarily involving the ovary and fallopian tubes. It is often misdiagnosed due to its nonspecific clinical presentation and radiological findings resembling malignancy [1]. This condition poses diagnostic challenges and may result in unnecessary extensive surgeries, leading to infertility and significant morbidity for affected individuals. Xanthogranulomatous inflammation has been predominantly described in extragenital organs such as the kidney, gallbladder, and urinary bladder. However, its occurrence in the female genital tract is relatively uncommon, but it is limited to the endometrium if found in the female genital tract [2]. The first documented case of xanthogranulomatous salpingo-oophoritis was reported by Gray and Libbey in 2001, highlighting the need for further understanding and awareness of this condition [3].

Several factors have been implicated in the pathogenesis of xanthogranulomatous inflammation, including pelvic inflammatory disease, abnormal lipid metabolism, and bacterial exposure. Ineffective antibiotic therapy, intrauterine contraceptive devices, and previous abdominal surgeries have also been associated with its development [4,5]. Histopathological examination remains the gold standard for diagnosing xanthogranulomatous inflammation, with characteristic findings of lipid-laden macrophages and chronic inflammatory cells. Differential diagnoses include ovarian malignancy, endometrioma, and tubo-ovarian abscess, necessitating comprehensive clinical evaluation and investigative workup [6].

Given the diagnostic challenges and potential for mismanagement, it is crucial to raise awareness among clinicians and pathologists regarding the clinical features, diagnostic modalities, and management strategies for xanthogranulomatous inflammation of the female genital tract.

## 2 Case presentation

The work has been reported in line with the SCARE criteria [7]. A 37-year-old female, gravida 4, para 4, was advised renal transplantation while on dialysis due to chronic kidney disease. A preoperative examination revealed a significant left adnexal mass. The patient presented with lower abdominal fullness, pain, and irregular menstrual cycles. Given the complexity of the patient's medical history and the ongoing renal transplantation process, radiological investigations were conducted, suggesting a tubo-ovarian abscess or neoplastic lesion.

A mass equivalent to a 20-week uterine size was palpated upon clinical examination. A gentle vaginal examination was attempted, but the cervix could not be felt due to the fullness of the pouch of Douglas, which was abutting the vagina and rectum, causing discomfort for the patient.

Haematological investigations revealed a haemoglobin level of 8.0 g%, a total leukocyte count of 14,000/mm3, and abnormalities in liver function tests (total bilirubin-1.22 mg/dl, GGT-178 U/l, ALP-424 U/l, with low albumin and proteins), as well as renal function tests (creatinine-2.45 mg/dl, urea- 45 mg/dl). The lipid profile was within normal limits, and tuberculosis screening was negative. Tumor marker CA-125 was elevated at 98 mg/dl, while other tumor markers were within normal limits. Table 1 shows the baseline workup of the patient.

**Table 1:** Baseline Lab Tests.

Lab test	Result	Normal range
Haemoglobin	8.0 g/dl	12.1 to 15.1 g/dl
Total leukocyte count	14,000 /mm3	4500 to 11,000 /mm3
Bilirubin	1.22 mg/dl	0.1 to 1.2 mg/dl
GGT(Gamma Glutamyl Transferase) blood test	178 IU/l	0 to 30 IU/l
ALP	424 IU/l	44 to 147 IU/l
Creatinine	2.45 mg/dl	0.6 to 1.1 mg/dl
Urea levels	45 mg/dl	5 to 20 mg/dl
CA-125 Marker	98 mg/dl	0 to 35 units/dl

**Fig. 1:**
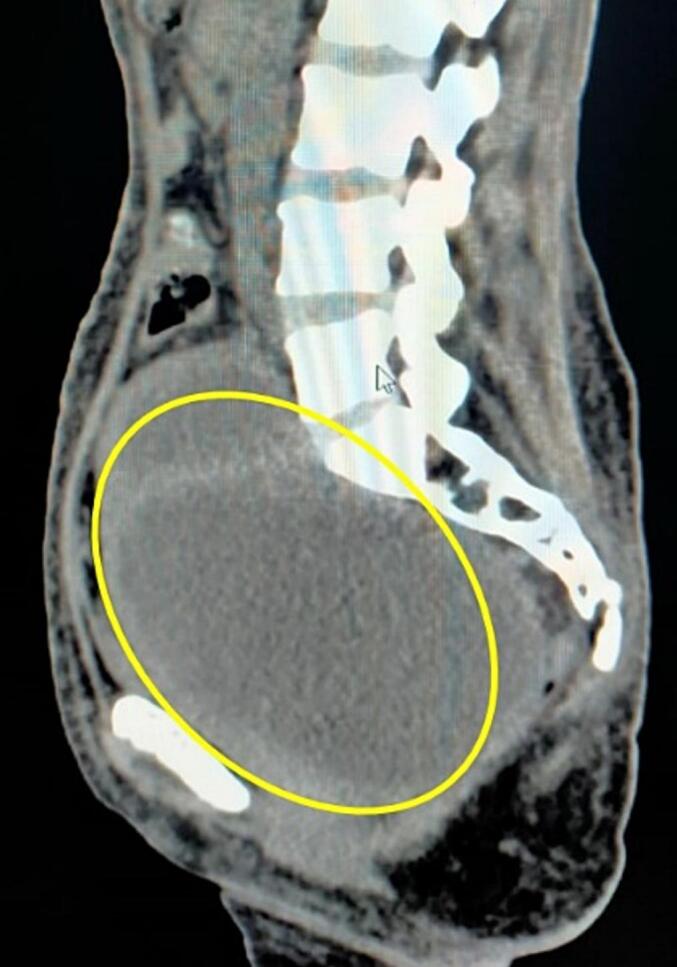
Ultrasonography (USG). Ultrasonography revealed a midline thick-walled adnexal cyst measuring 11 × 10 cm with internal septation.

**Fig. 2:**
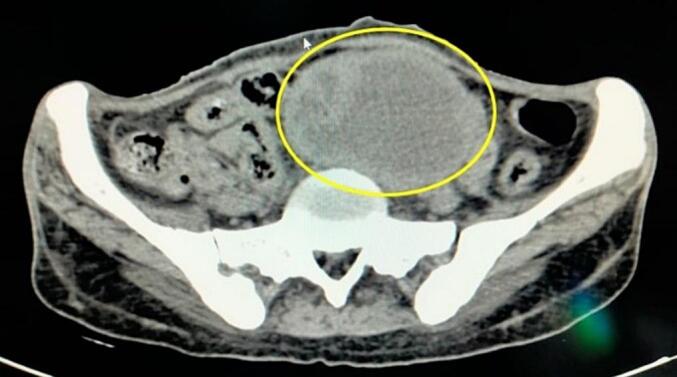
CT-Scan abdomen. CT imaging demonstrated a large left adnexal thick-walled, multiloculated, incomplete septations of 12 × 10 × 11cm, suggestive of a tubo-ovarian abscess or neoplastic lesion.

Ultrasonography (USG) revealed a midline thick-walled adnexal cyst measuring 11 cm × 10 cm with internal septation, and the contralateral ovary was not visualized. The uterus appeared normal in size **(**Fig. 1**)**. CT imaging demonstrated a large left adnexal thick-walled, multiloculated, incomplete septations of 12 cm × 10 cm × 11 cm, suggestive of a tubo-ovarian abscess or neoplastic lesion **(**Fig. 2**)**.

**Fig. 3:**
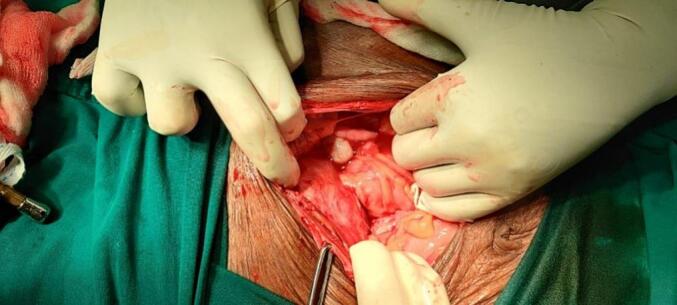
Adherent left tubo-ovarian mass observed during laparotomy.

**Fig. 4:**
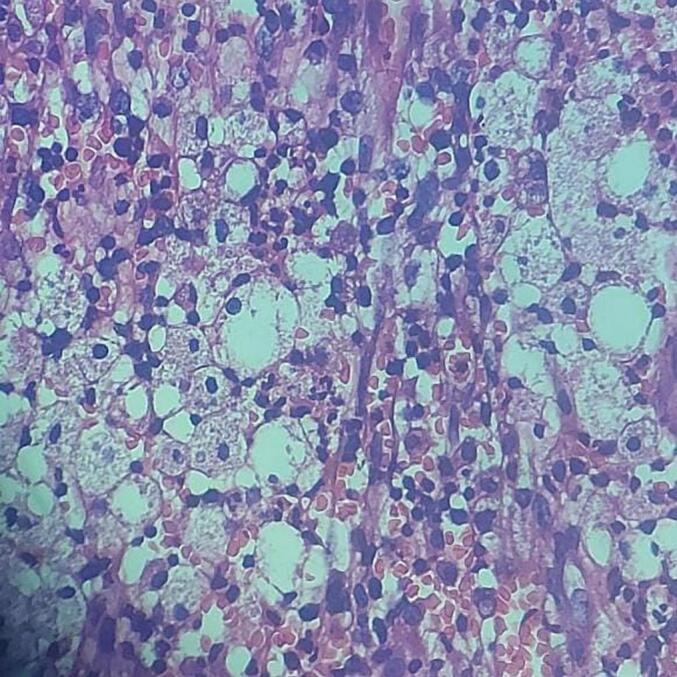
Histopathologic imaging showing the presence of lipid-laden macrophages and chronic inflammatory cells.

During staging laparotomy, the left tubo-ovarian mass was found to be adherent to the urinary bladder, anterior wall of the uterus, and bowel, making further dissection challenging **(**Fig. 3**)**. Accessible tissue was sent for frozen section analysis, which indicated a benign lesion, leading to the deferral of hysterectomy. The resected tubo-ovarian mass and pus culture were sent for histopathological evaluation **(**Fig. 4**)**. The final diagnosis was confirmed as xanthogranulomatosis salpingo-oophoritis. Culture sensitivity testing was conducted to guide appropriate treatment. Post-operative recovery was uneventful.

## 3 Discussion

Xanthogranulomatous inflammation of the female genital tract, although rare, presents significant diagnostic challenges and therapeutic dilemma [5,8]. In 1976, the first case of xanthogranulomatous inflammation was described by Kunakemakorn et al. of serosa of uterus, left fallopian tube, and ovary in his report of inflammatory pseudotumor in the pelvis [9]. The case highlights the intricate management considerations in diagnosing and treating xanthogranulomatous salpingo-oophoritis in a patient undergoing renal transplantation while on dialysis. The importance of a comprehensive evaluation, thorough clinical evaluation, meticulous radiological assessment, and histopathological examination, accurate diagnosis, and careful decision-making in the context of renal failure and transplantation is emphasized.

The pathogenesis of xanthogranulomatous inflammation remains incompletely understood, with various predisposing factors implicated in its development [10,11]. These factors include pelvic inflammatory disease, abnormal lipid metabolism, bacterial exposure, and previous abdominal surgeries [4,5]. In the present case, the patient's medical history, including chronic kidney disease requiring dialysis and a previously treated episode of pulmonary tuberculosis, may have contributed to her predisposition to xanthogranulomatous salpingo-oophoritis [12].

Histopathological examination is crucial in confirming the diagnosis of xanthogranulomatous inflammation [13]. Characteristic findings include the presence of lipid-laden macrophages and chronic inflammatory cells within affected tissues. Differential diagnoses, such as ovarian malignancy, endometrioma, and tubo-ovarian abscess, must be carefully considered and excluded through comprehensive clinical and histopathological evaluation [14]. Additionally, malakoplakia is one of the differentials. Malakoplakia and xanthogranulomatous inflammation were identical chronic inflammatory diseases but the former one has Michaelis-Gutmann bodies (basophilic cytoplasmic concentric calcific bodies inside histiocytes), which are absent in xanthogranulomatous inflammation [15].

Managing xanthogranulomatous inflammation typically involves surgical intervention, with oophorectomy being the preferred treatment modality [16]. However, misdiagnosis may lead to unnecessary extensive surgeries, including hysterectomy, resulting in infertility and significant morbidity for affected individuals [17]. Therefore, accurate preoperative diagnosis and intraoperative assessment are imperative to avoid overtreatment and preserve fertility whenever possible [18]. In the case presented, staging laparotomy was performed due to the suspicion of malignancy based on clinical and radiological findings. Intraoperatively, the challenging dissection due to adherence of the tubo-ovarian mass to adjacent structures underscored the complexity of managing xanthogranulomatous inflammation. [Fig. 1] Frozen section analysis was crucial in guiding surgical decision-making, ultimately leading to the deferral of hysterectomy in favor of organ preservation. Further research is warranted to elucidate the underlying mechanisms and risk factors associated with xanthogranulomatous inflammation of the female genital tract. Additionally, prospective studies evaluating the long-term outcomes and fertility preservation strategies in affected individuals are needed to optimize patient care and improve clinical outcomes.

## 4 Conclusion

This case report underscores the need for careful diagnostic evaluation in patients with vague pelvic symptoms. Despite its rarity, accurate diagnosis of xanthogranulomatosis salpingo-oophoritis is crucial to avoid unnecessary surgeries and preserve fertility. A definitive diagnosis was established through comprehensive assessment and meticulous histopathological analysis, guiding appropriate surgical intervention. With the patient's post-operative recovery progressing well, ongoing monitoring and management are essential.

## Author contribution

Conceptualization: Mahalakshmi Gaddi, Vidya Kamat

Investigation: Sahana Surpur

Methodology: Vidya Kamat, Mahalakshmi Gaddi

Project Administration: Gaurav Mittal

Supervision: Mayur Wanjari, Gaurav Mittal Mittal

Writing the original draft: Zoha Khan, Gaurav Mittal, Sahana Surpur

## Consent

Consent was waived from the patient before intervention and publication.

## Ethical approval

Since this was a case report no ethical approval was required for this article.

## Guarantor

Mahalakshmi Gaddi and Gaurav Mittal accept full responsibility of the work and act as the Guarantor.

## Sources of funding

None.

## Declaration of competing interest

The authors declare no conflicts of interest.
